# Self-medication Practice of Antibiotics among Medical and Dental Undergraduate Students in a Medical College in Eastern Nepal: A Descriptive Cross-sectional Study

**DOI:** 10.31729/jnma.4914

**Published:** 2020-05

**Authors:** Namita Kumari Mandal, Gajendra Prasad Rauniyar, Dilli Sher Rai, Dipesh Raj Panday, Ramayan Prasad Kushwaha, Santosh Kumari Agrawal, Pragya Regmee

**Affiliations:** 1 Department of Pharmacology, BP Koirala Institute of Health Sciences, Dharan, Nepal; 2 Department of Public Health Dentistry, College of Dental Surgery, BP Koirala Institute of Health Sciences, Dharan, Nepal; 3 Department of Oral Medicine and Radiology, BP Koirala Institute of Health Sciences, Dharan, Nepal

**Keywords:** *antibiotics*, *drugs*, *medical students*, *prescriptions*, *self-medicatio*

## Abstract

**Introduction:**

Self-medication plays significant role in the development of adverse drug reactions, antibiotic resistance, and masking of underlying diseases. Medical students have some knowledge about the use of antibiotics and have a higher chance of irrational and injudicious use. This study aims to find the prevalence of self-medication practice of antibiotics among medical and dental undergraduate students.

**Methods:**

A descriptive cross-sectional study was done among medical and dental undergraduate students from the first year to the fifth year at BP Koirala Institute of Health Sciences from 1st June 2018 to 30th August 2018. Ethical approval was obtained from the Institutional Review Committee (IRC/1210/018). Whole sampling was done. Data was collected using a self-responding, semi-structured questionnaire and analyzed using Statistical Package for the Social Sciences version 11.5.

**Results:**

In total 558 students, the prevalence of self-medication practice of different antibiotics was 285 (51.1%) within the past year. Among self-medicated students, 152 (53.3%) were males. The common drug self-medicated was Azithromycin 80 (28.1%) and the common medical condition to use non-prescription antibiotics was for treatment of sore throat with runny nose 129 (45.3%). The main source for obtaining non-prescription antibiotics were retail pharmacies 157 (55.1%).

**Conclusions:**

Self-medication with antibiotics was at increasing rate with each succeeding years of the medical courses. Medical students should be made aware of the rational use of antibiotics by incorporating appropriate courses in their academic curriculum for more refined practice on antibiotics rather than advancement of theoretical knowledge alone.

## INTRODUCTION

Antibiotic resistance is a growing health concern worldwide.^1,2^ Irrational and injudicious use of antibiotic drugs has led to the emergence of antibiotic-resistant pathogens that imposes a constant threat to the existing health care system. It produces a huge economic burden to the country.^3,4^

Research evidence suggests, the self-medication practice of antibiotics is increasing worldwide.^5-7^ The medical students have better access to healthcare-related information and facilities. The practice of rational use of antibiotics during study periods may have a positive impact on the future use of the antibiotics.^8^ Therefore, this survey is very important to determine the self-medication practice of antibiotics among medical and dental students to prevent the irrational use of antibiotics and to promote safe medication practice.

The main aim of this study is to find out the prevalence of self-medication practice of antibiotic among the undergraduate medical and dental students at BP Koirala Institute of Health Sciences.

## METHODS

This descriptive cross-sectional study was conducted from 1^st^ June 2018 to 30^th^ August 2018 among medical and dental undergraduate students at BP Koirala Institute of Health Sciences (BPKIHS), Dharan, Nepal. Ethical approval was obtained from the Institutional Review Committee of BPKIHS (IRC/1210/018). Before the commencement of the study, the students were explained about the objectives of the study and the written informed consent was obtained. Inclusion criteria constituted all the medical and dental undergraduate students from the first year to the fifth year. Those students who were non-consenting were excluded from the study. The confidentiality and anonymity of the students were maintained.

The data were collected using a self-prepared questionnaire, which was finalized after pre-testing on 18 students (10% of the study sample) and necessary modifications were made in the questionnaire. The students who had participated in the pre-testing were not included in the main study. To increase the response rate appreciably, students sitting in Lecture Theater were approached after their morning classes. The whole sampling method was applied and the entire population of medical and dental students present at the time of data collection was considered in this study.

The semi-structured questionnaire was divided into two parts. The first part contained questions on demographic profiles: age, gender, the academic year of study, and the parent’s occupation. The second part contained questions regarding self-medication practice of antibiotic use within one year. The questions were mainly focused on the types of self-medicated antibiotics, the place of obtaining non-prescription antibiotics, the reason for self-medication, and any adverse effects experienced during self-medication. Students were encouraged to answer all the questions.

The obtained data was carefully analyzed using the Statistical Package for the Social Sciences (SPSS) version 11.5. The descriptive parameters like mean, frequency, standard deviation, and percent were calculated and tabulated.

## RESULTS

**Figure 1:**
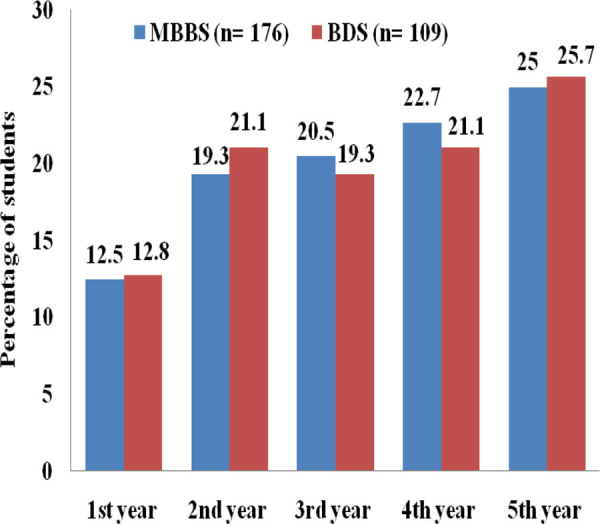
Study the year-wise distribution of self-medication of antibiotics.

In a total of 558 students, the prevalence of self-medication practice of different antibiotics was 285 (51.1%) within the past year. The self-medication with antibiotics was seen more in medical students 176 (61.7%) than dental students 109 (38.3%). Among medical and dental students, fifth-year students were used more antibiotics than the rest of the study year students (Figure 1).

**Figure 2:**
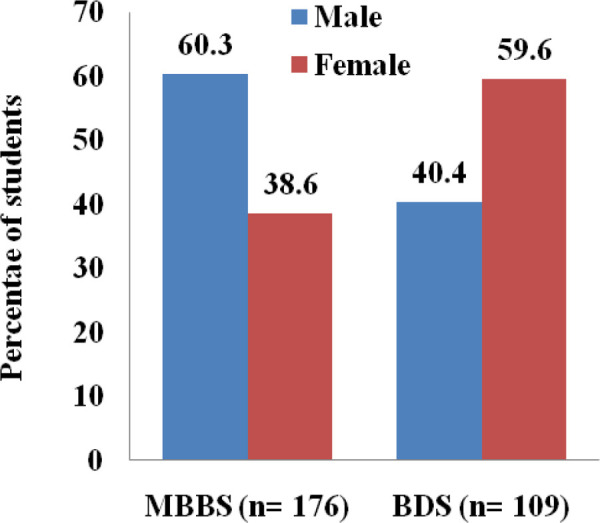
Sex wise distribution of self-medication of antibiotics.

The age of the students who had self-medicated with an antibiotic ranged from 19-27 years with a mean age of 22.6±1.8. Overall, male students 152 (53.3%) were used more self-medicated antibiotics than female 133 (46.7%) students (Figure 2).

**Figure 3:**
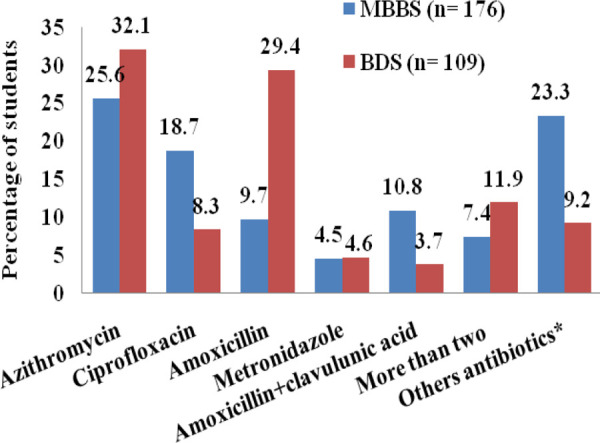
Types of self-medicated antibiotics.

Among 285 students. Azithromycin 80 (28.1%), Ciprofloxacin 42 (14.7%), Amoxicillin 49 (17.2%), and Amoxicillin+Clavulunic acids 13 (4.6%) were the common self-medicated antibiotics. However, Azithromycin 45 (25.6%) and Ciprofloxacin 33 (18.7%) were more common in medical students, and Azithromycin 35 (32.1%) and Amoxicillin 32 (29.4%) were in dental students (Figure 3).

*Other antibiotics include Cefelexin, Cefixime, Cefadroxil, Clindamycin, Itraconazole, Levofloxacin, Mupirocine, Seconidazole, Ofloxacin.

**Figure 4:**
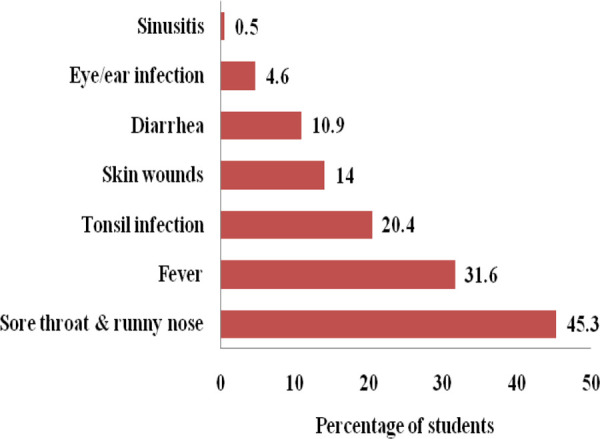
The medical condition for which self-medication of antibiotics (n= 285).

The common medical conditions for which most of the participants from both the discipline had self-medicated with antibiotics were for the treatment of sore throat with runny nose 129 (45.3%) followed by fever 90 (31.6%), tonsil infection 58 (20.4%), and skin wounds 40 (14.0%) (Figure 4).

**Figure 5:**
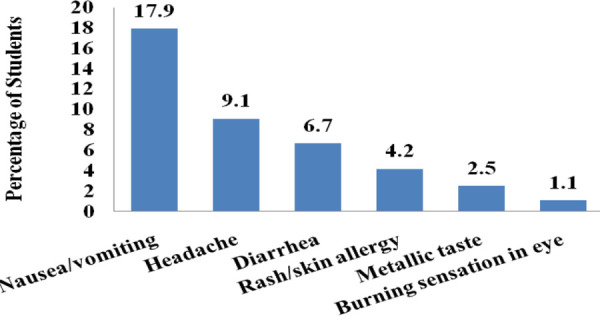
Adverse effects experienced after self-medication of antibiotics (n= 285).

The common adverse effects experienced by the students during self-medication were nausea or vomiting 51 (17.9%), followed by headache 26 (9.1%), diarrhea 19 (6.7%), and rash or skin allergy 12 (4.2%) (Figure 5).

More than half 157 (55.1%) of the participants had obtained non-prescription antibiotics from the retail pharmacies. Fifty-one (17.9%) students obtained is from their family members or relatives and 49 (17.2%) had used leftover medicines from the previous prescriptions. Similarly, 50 (17.5%) students obtained antibiotics from their friends or seniors and only 3 (1.1%) students obtained from medical representatives.

## DISCUSSION

The overall prevalence of self-medication was 51.1% among undergraduate students from both the medical and dental streams at BP Koirala Institute of Health Sciences. A study by Sarraf et al.^9^ in the same setting in 2015, the prevalence of self-medication was 19.2% among medical and dental undergraduate students. Similarly, Pant et al.^5^ Study in 2015 among dental undergraduate students at Nepal Medical College Teaching Hospital, the prevalence of self-medication was 35.1%. In other studies conducted worldwide, Sri Lanka (40.9%),^6^China (75.3%),^7^Jordan (63.9%),^10^ Ethiopia (39.7%),^11^ and Nigeria (38.8%),^12^ there was a widespread presence of self-medication among medical students. The variation in prevalence may be due to the differences in the selection of study populations, easy availability of antibiotics, and the socio-economic condition of the countries. At the same time, it also signifies there is no decreasing trend of self-medication among medical students.

In the studies done by Ratish et al,^6^ and Huang et al,^7^ it was seen that percentage of self-medication with antibiotics gradually increased with the increase of each study year in the medical institution. These findings are consistent with our study. It is explained as the students gain more knowledge in medicine with each progressing year and build up enough confidence to treat medical conditions. It can naturally prompt students in managing minor illness by themselves and their friends, though they do not have the right to prescribe to others until they become a certified prescriber.^6^

In our study retail pharmacies were the main source of non-prescription antibiotics. However, some students also used leftover medicines and procuring antibiotics from friends and relatives. Similarly, Ratishetal^6^ study showed, retail pharmacies, relatives, friends and remaining medicine in the household were the main source of non-prescription antibiotics. In developing countries, many drugs are easily available without a registered doctor’s prescription. Even parent, relatives, friend easily gets medicine from retail pharmacies due to poor drug selling regulations and the lack of awareness about drug use, side effects, resistance, and drug interaction. Medical students have some knowledge regarding antibiotics and do not want to visit a doctor until and unless some severe presentation of the disease or complications. These factors promote the students to use self-medication of antibiotics.

Previous studies conducted in Nepal, India, Sri Lanka, and China, showed that the common cold was the most common medical condition for which medical students were self-medicated the antibiotics.^5-9^ In our study, it was seen that Azithromycin (31.8%) followed by Amoxicillin (21.1%) was the most common antibiotic used for self-medication. Azithromycin has a special property like the incidence of fewer side effects, and intake of single daily oral dose for a minimum of three days is sufficient to treat most of the respiratory bacterial infection.^13^,^14^ However, other studies showed Amoxicillin was a predominantly self-medicated drug.^6^,^7^,^10^,^15^ These both antibiotics are broad-spectrum and effective against a wide range of microbes. Injudicious use of broad-spectrum antibiotics for treating trivial infections or viral infections like a common cold where it is ineffective may lead to the development of multi-drug resistant bacteria.^16^ The infection with antibiotic-resistant organisms is very difficult to treat with available first and second-line drugs and may force the health professionals to use more toxic and expensive antibiotics.^17^ It may lead to a more economic burden not only to the existing health care system of the country but also to the patients and their families.^18^ This malpractice should be promptly stopped. Therefore, medical students should be repeatedly made aware of the rational use of the antibiotics by properly incorporating appropriate courses in their academic curriculum focusing more on refined practice on antibiotics rather than the advance of knowledge alone.

The findings of this study cannot establish the causal association and couldn't be generalized because it was conducted in a single medical institution only. A nationwide study, including all medical institutes in Nepal, would be considered. Chance of recall bias cannot be ruled out as participants were asked to remember about the incidence of the past one year.

## CONCLUSIONS

This study revealed the presence of self-medication with antibiotics is increasing with succeeding years of the medical course. Medical students should be repeatedly made aware of the rational use of antibiotics by properly incorporating appropriate courses in their academic curriculum for more refined practice on antibiotics rather than the advancement of theoretical knowledge alone.
